# Exclusive breastfeeding and risk of malnutrition in children: the healthcare provider-led education in Mwanza, Tanzania

**DOI:** 10.3389/fnut.2026.1793685

**Published:** 2026-06-22

**Authors:** Joel Mwanga, Farida Iddy Mkassy, Titus Robert Leeyio, Stella Mugassa, Ednah Loishiye Samwel, Mirriam Dianah Lucheveleli, Janeth Peter, Paul Alikado Sabuni, Msilikale Walter Manyiri, Peter Martin Chilipweli, Eveline Thobias Konje, Martha Fidelis Mushi

**Affiliations:** 1 Department of Clinical Nursing and Research, Archbishop Mayala School of Nursing, Catholic University of Health and Allied Sciences (CUHAS), Mwanza, Tanzania; 2 Department of Epidemiology, Biostatistics, and Behavioral Science, School of Public Health, Catholic University of Health and Allied Sciences (CUHAS), Mwanza, Tanzania; 3 Faculty of Science, Hasselt University, Diepenbeek, Belgium; 4 Biomedical Research and Clinical Trials Department, Ifakara Health Institute, Dar es Salaam, Tanzania; 5 Household Economics and Health System Research Unit, Department of Epidemiology and Public Health, Swiss Tropical and Public Health Institute, Basel, Switzerland; 6 Department of Community Medicine, School of Public Health, Catholic University of Health and Allied Sciences (CUHAS), Mwanza, Tanzania; 7 Department of Microbiology and Immunology, Weill Bugando School of Medicine, Catholic University of Health and Allied Sciences (CUHAS), Mwanza, Tanzania

**Keywords:** Bayesian logistic regression, child malnutrition, exclusive breastfeeding, healthcare provider, maternal practices, postnatal care

## Abstract

**Background:**

Malnutrition continues to pose a significant threat to children’s survival, growth, and development. It manifests in various forms, including stunting, wasting, overweight, and underweight. Furthermore, exclusive breastfeeding protects children from gastrointestinal infections and promotes healthy weight gain, which in turn helps reduce the risk of malnutrition. Healthcare providers are crucial in the prevention & reduction of malnutrition by providing ongoing support during the maternal sequence of care and through the provision of nutritional health-related education.

**Objective:**

This study aims to determine the relationship between exclusive breastfeeding and malnutrition, and to triangulate healthcare providers’ practice in delivering health education to reduce malnutrition.

**Methodology:**

Five hundred mothers with their children aged 6–24 months and 15 healthcare providers were recruited for the study. Bayesian logistic regression with three Markov Chain Monte Carlo (MCMC), 10,000 iterations, and non-informative priors was performed to assess the association between exclusive breastfeeding and malnutrition, with 95% credible intervals used to assess statistical significance.

**Result:**

In this study, only 18% of mothers reported exclusive breastfeeding in the first 6 months, and the overall prevalence of malnutrition was 27.8%. After adjusting for marital status and the mother’s education, we obtained the non-significant lower odds of malnutrition among exclusively breastfed children compared to babies who are not exclusively breastfed (OR = 0.9, 95% CI:0.7–1.1). Based on health education sessions observed, healthcare providers rarely discussed breastfeeding position, exclusive breastfeeding, food and general hygiene, and early recognition of danger signs among children. Overwhelming workload and shortage of staff were common challenges mentioned by healthcare providers.

**Conclusion:**

In this study, more than a quarter of children aged 6–24 months were found to have malnutrition, with exclusive breastfeeding being a rare practice among participants. This highlights the need for continuous feeding support through nutritional education sessions to promote exclusive breastfeeding among postnatal women. However, limited nutritional education talks on nutrition, breastfeeding, hygiene, and recognition of early signs of illness were provided by healthcare providers due to reported health system operational challenges. This could explain the gap observed among postnatal mothers on exclusive breastfeeding practice, hygiene, and recognition of early signs of illness.

## 1 Introduction

Malnutrition continues to pose a significant threat to children’s survival, growth, and development, manifesting in various forms, including stunting, wasting, overweight, and underweight. According to WHO data from 2019 and 2022, the prevalence of stunting increased slightly from 21.3 to 22.3%, wasting decreased marginally from 6.9 to 6.8%, and the prevalence of overweight remained constant at 5.6% globally (1, 2). In response to the global burden of malnutrition, the United Nations incorporated nutrition improvement into the Sustainable Development Goals (SDGs). Specifically, SDG 2 aims to end hunger, achieve food security, improve nutrition, and promote sustainable agriculture, with target 2.2 emphasizing ending all forms of malnutrition for children under 5 years (3). Despite these commitments, many countries continue to face challenges in reducing child malnutrition. In Tanzania, malnutrition remains a major public health concern. According to the 2022 Tanzania Demographic and Health Survey, the prevalence of stunting and underweight is estimated to be 30 and 12%, respectively (4). Furthermore, the 2022 SDG report indicates that Tanzania achieved about 52% of the targets for SDG 2. While progress has been made in reducing wasting and overweight, stunting and underweight remain persistent forms of malnutrition (3). This shows the need for protective practices, with exclusive breastfeeding recognized as one of the most effective strategies.

Exclusive breastfeeding is widely acknowledged as a key protective practice against malnutrition. It involves feeding an infant solely with breast milk, without additional food or liquids other than vitamin supplements or prescribed medications (1). This practice delivers all essential nutrients in the correct proportions and provides immune-protective factors that enhance nutrient absorption and prevent infections, thereby improving the child’s nutritional status (5). In addition, exclusive breastfeeding supports healthier habits and promotes appropriate weight gain, which further reduces the risk of malnutrition (6, 7). Despite these well-established benefits, global prevalence remains low at only (32%) (8). Due to the fact that exclusive breastfeeding requires knowledge, skills, and ongoing support, understanding the role of healthcare providers in promoting and sustaining this practice is essential.

Healthcare providers leading health education can reshape caregivers’ feeding practices to promote exclusive breastfeeding and reduce malnutrition. They provide key support through postnatal education on feeding, hygiene, and early signs of malnutrition, which equip mothers with the knowledge and skills necessary to improve child health (9). Through personalized, one-on-one counseling, providers address caregivers’ concerns, such as perceived low milk supply, emphasize the benefits of exclusive breastfeeding, and build maternal confidence, thereby fostering adherence to recommended infant feeding practices (10, 11). This strong influence contributes to higher exclusive breastfeeding rates, which in turn reduces the risk of child malnutrition. However, despite this critical role, evidence from Tanzania on the impact of provider-led interventions remains limited, and challenges such as staff shortage and inadequate training can further restrict their effectiveness (12). This persistence of malnutrition highlights the need to understand not only caregiver practices but also the actual nutritional outcomes among children.

Several studies have reported the prevalence of malnutrition, specifically wasting, stunting, and underweight, across various regions in Tanzania, with rates exceeding 20% (13–15). These findings show that malnutrition remains a significant public health concern since it weakens the immune system of the children, which consequently causes death and increases the mortality rate (16). While previous studies focused on individuals forms and association with breastfeeding, triangulating with health education support offered by health workers remains limited. By triangulating these three data sources, this study aims to provide a more comprehensive understanding of how provider-led education influences caregiver feeding practices and child nutritional outcomes, thereby offering actionable evidence for targeted interventions in Tanzania. Building on this gap, this study aims to assess the extent and patterns of malnutrition among children under 2 years of age, including wasting, underweight, stunting, and overweight. It also examines the relationship between exclusive breastfeeding and malnutrition while adjusting for potential confounding factors, and explores healthcare provider practices in delivering nutrition-related health education. Collectively, the study seeks to generate evidence to improve the nutritional status and well-being of children under 2 years of age.

## 2 Materials and methodology

### 2.1 Study setting

A cross-sectional study was conducted in the pre-selected reproductive and child health unit (RCH) of public health facilities in Mwanza City, North-Western Tanzania. In Mwanza city, there are two districts, Ilemela and Nyamagana, with 39 public health facilities. This study focused on facilities with a high volume of attendance for pregnant and postnatal women (17–19). Hence, the study was conducted at Nyamagana District Hospital, Makongoro Health Center, and Buzuruga Health Center. The selected health facilities provide basic emergency obstetric and newborn care (BEmOC) and comprehensive emergency obstetric and newborn care (CEmOC) services. Along with prenatal, postnatal, and education services, these three clinics offer vaccinations, reproductive health care, and assistance to mothers and their children.

### 2.2 Study population

The study involved health care providers and mothers/caregivers of children aged 6–24 months attending at Nyamagana District Hospital, Makongoro RCH, and Buzuruga HC who were willing to participate in the study from March 2024 to May 2024. All health care providers who were actively providing services at the RCH unit and mothers/caregivers of children aged 6–24 months who were attending RCH clinics for child health service postnatally were included. The study excluded all health care providers who were away from the workstation during the data collection period and mothers/caregivers whose babies attended the study sites for seeking medical attention, besides services provided during postnatal sessions.

### 2.3 Sample size and sampling procedure

Fifteen health education sessions were observed in three preselected study sites of which each participating healthcare provider was observed once. All available health care providers were recruited through full enumeration due to the relatively small number of staff working at the RCH unit. The sample size for the mothers/caregivers was calculated using the Kish-Leslie formula 1965 (20) which gives the total sample of 354, taking into account 95% confidence level, a prevalence of stunting of 0.36 (21), and a margin of error of 0.05. Considering the design effect of 1.41, the required sample size for this study was 500 participants (mothers/caregivers). However, due to a lack of sampling frame, and clients would come and go after receiving postnatal care services, all mothers who attended postnatal clinics with their infants aged 6–24 months and volunteered willingly to participate were invited to the study. Using convenient sampling was feasible from practicability point of view. Although the sample size was calculated to ensure adequate precision for estimating the prevalence of the outcome of interest, selecting participants using a probability sampling technique would not be possible, hence a correction factor of 1.41 was utilized.

### 2.4 Data collection procedure

The structured checklist was designed to capture healthcare provider practices during health education sessions, focusing on both actions and topics covered based on postnatal care guidelines on nutrition topics and teaching materials. Although healthcare providers were informed that observations would take place, they were not told the exact day for observations, and no identity for the observer was disclosed to reduce the chance of behavior change during health education sessions. The observation was followed by a brief structured questionnaire administered to healthcare providers to document challenges encountered, their willingness to engage with mothers/caregivers at the end of the day, specifically when they were done with the provision of postnatal care services. The exit structured questionnaire, which consisted of women’s practices regarding proper infant feeding, including breastfeeding, hygiene, food preparation, and early recognition of malnutrition, was developed by the authors after involving experts from maternal and child health units and postnatal care guidelines. The tools were pilot-tested on 50 mothers/caregivers and 10 healthcare providers, respectively, in different sites, to double-check the clarity, feasibility, and accuracy of measurement procedures before full data collection. Additionally, the anthropometric measurements (height, weight, and mid-upper arm circumference) of children were captured as part of standard care provided during postnatal care using a weighing scale, stadiometer, and MUAC tape available at the health facility. All tools were developed in English and subsequently translated into Swahili. To improve and maintain data quality, the use of a structured checklist, researcher assistants’ training, pilot testing, and multiple use of observers were considered throughout the data collection period.

### 2.5 Data management and analysis procedure

Data was entered into Microsoft Excel (version 2016) and transferred to R version 4.1.3. Simple descriptive statistics were performed to obtain the socio-demographic characteristics of study participants. Prevalence of stunting (height-for-age Z-score *< −*2), underweight (weight-for-age Z-score *< −*2), overweight (weight-for-age Z-score *>* 2), and wasting (weight-for-height Z-score *< −*2) were estimated. The malnutrition prevalence was estimated by combining all four indicators (stunting, overweight, underweight, and wasting) for children with at least one of them. The composite outcome was used to capture the overall burden of malnutrition, as children may experience multiple forms simultaneously, providing a more comprehensive measure of nutritional risk.

The association between exclusive breastfeeding and malnutrition was evaluated using Bayesian binary logistic regression (BBLR) with non-informative priors following a normal distribution, employing 3 Markov Chain Monte Carlo (MCMC) chains. The significance of the result was assessed by the 95% credible intervals. The model fit for the BBLR was assessed by the use of traceplots, density plots, and Gelman plots for parameters.

BBLR was employed because the outcome variable (malnutrition) was binary, making logistic regression an appropriate modeling approach for assessing associations in cross-sectional data. The Bayesian framework was preferred as it allows direct probabilistic interpretation of parameter estimates through credible intervals (22). A 95% credible interval represents the probability that the true parameter value lies within the interval, given the observed data and the specified prior distribution. In addition, Bayesian methods provide more stable estimates compared to frequentist approaches, particularly in the presence of sparse data or small sample sizes, which further supports their use in this study.

The mathematical expression for the BBLR model is as follows;


$$\text{Likelihood}\;(L);Y_{i}|\pi_{i}∼\text{Bernoulli}\;(\pi_{i}),i=1,\ldots ,N$$

$$\text{logit}(\pi_{i})=\beta_{0}+\beta_{1}X_{1i}+\ldots +\beta_{p}X_{\mathrm{pi}}$$

Priors;


$$\beta_{j}\sim \text{Normal}(0,\sigma^{2}),j=0,1,\ldots ,p$$

Posterior;


$$p(\beta_{0},\ldots ,\beta_{p})\alpha \;\prod_{i=1}^{N}{\pi_{i}}^{Y_{i}}{\left(1-\pi_{i}\right)}^{1-Y_{i}}.\prod_{j=0}^{p}\frac{1}{\sqrt{2\pi \sigma^{2}}}\;\exp(-\frac{\beta_{j}^{2}}{2\sigma^{2}}\;)$$

Where *i* is the observation, *p* is the number of predictor variable, *X* is the predictor, *β* is the parameter, and *L* is the likelihood, which goes together with our fitted model.

Potential confounders were identified through a review of the literature and frequentist binary logistic regression. A variable was considered a confounder if including it in the model changed the estimated association between exclusive breastfeeding and malnutrition by more than 10% using binary logistic regression. Effect modification was tested using the Mantel–Haenszel test, which examines the null hypothesis of homogeneity. In this study, the variables tested for both confounding and modification were the mother’s age, the mother’s education, antenatal clinic attendance, the mother’s marital status, and the mother’s occupation.

A Directed Acyclic Graph (DAG) was used to represent the relationship between exclusive breastfeeding and malnutrition and to identify confounders for adjustments to avoid over-adjustment and improve the validity of the estimated association. The DAG was constructed using DAGitty.

### 2.6 Ethical consideration

Ethical clearance for this study was obtained from the joint CUHAC/BMC ethical committee with registration number BMC/CUHAS/CREC/2904/2024. Written informed consent was obtained from all participants. The participant’s information was handled confidentially, and the questionnaire did not contain their names but their ID number.

## 3 Results

### 3.1 Demographic, WASH, maternal and healthcare characteristics

The study involved 500 mother–child pairs who were attending postnatal care services at three preselected public health facilities (see Table 1). The majority of mothers were married (81.8%), had primary/secondary education level (80.6%) with all most all deliveries occurred in health facilities (98.8%). Other hygiene related practices were commonly reported; boiling drinking water (70.8%), washing utensils (67.2%), but only one third of participants reported washing their breasts before breastfeeding (32.4%). For children who participated in the study nearly equally distributed by sex, two thirds (62.4%) were infants, and majority of them (88.6%) were born with normal weight.

**Table 1:** Demographic, maternal and healthcare, wash characteristics of postnatal women.

Characteristics	Variable	Category	*N* (%)
Demographic	Marital status	Currently married	409 (81.8)
		Currently not married	91 (18.2)
	Education level	No school	71 (14.2)
		Primary	223 (44.6)
		Secondary	180 (36.0)
		Tertiary	26 (5.2)
Maternal and healthcare	ANC attendance	Mean ± SD	4 ± 1.0
	Place of delivery	Home	3 (0.6)
		Roadside	3 (0.6)
		Health center	494 (98.8)
	Health facility attended	Nyamagana	201 (40.2)
		Makongoro	99 (19.8)
		Buzuruga	200 (40.0)
WASH	Boil drink water	Yes	354 (70.8)
		No	146 (29.2)
	Wash utensils	Yes	336 (67.2)
		No	164 (32.8)
	Wash breasts before breastfeeding	Yes	162 (32.4)
		No	338 (67.6)
	Food storage	Fridge	3 (0.6)
		Hot pan	362 (72.4)
		Plate/cup	135 (27.0)
Child’s Characteristics	Sex	Male	246 (49.2)
		Female	254 (50.8)
	Age group	6–12 months	312 (62.4)
		13–24 months	188 (37.6)
	Birthweight	< 2.5 Kg	56 (11.2)
		> = 2.5 Kg	444 (88.8)

#### 3.1.1 Malnutrition status and exclusive breastfeeding practices for the first 6 months

Overall prevalence of malnutrition estimated to be 27.8% (95% CI: 23.9–31.9%) with stunting (16.4, 95% CI: 13.3–20.0%) being the common malnutrition status (see Table 2). Breastfeeding within 24 h of life was reported by majority of women (96.6%) whereas exclusive breastfeeding was rarely practiced (18.0%) within the first 6 month of child’s life. Commonest food/drinks given to children within the first 6 months of life reported to be drinking water (60.6%), soft porridge (54.2%), and tea/juice (47.2%).

**Table 2:** Malnutrition status and breastfeeding practices in the first 6 months.

Characteristics	Variable	Category	*N* (%)
Malnutrition	Wasting	Yes	39 (7.8)
		No	461 (92.2)
	Underweight	Yes	18 (3.6)
		No	482 (96.4)
	Stunting	Yes	82 (16.4)
		No	418 (83.6)
	Overweight	Yes	20 (4.0)
		No	480 (96.0)
	Malnutrition	Yes	139 (27.8)
		No	361 (72.2)
Feeding practices	Breastfeeding within 24 h after birth	Yes	483 (96.6)
		No	17 (3.4)
	Giving water in the first 24 h after birth	Yes	28 (5.6)
		No	472 (94.4)
	Exclusively breastfeeding	Yes	90 (18.0)
		No	410 (82.0)
	Common food/drinks given in the first 6 months of life	Water with sugar/glucose	303 (60.6)
		Soft porridge	271 (54.2)
		Tea or juice	236 (47.2)
		Cow milk	142 (28.4)
		Formula milk	75 (15.0)

### 3.2 Association between exclusive breastfeeding and malnutrition

**Figure 1:**
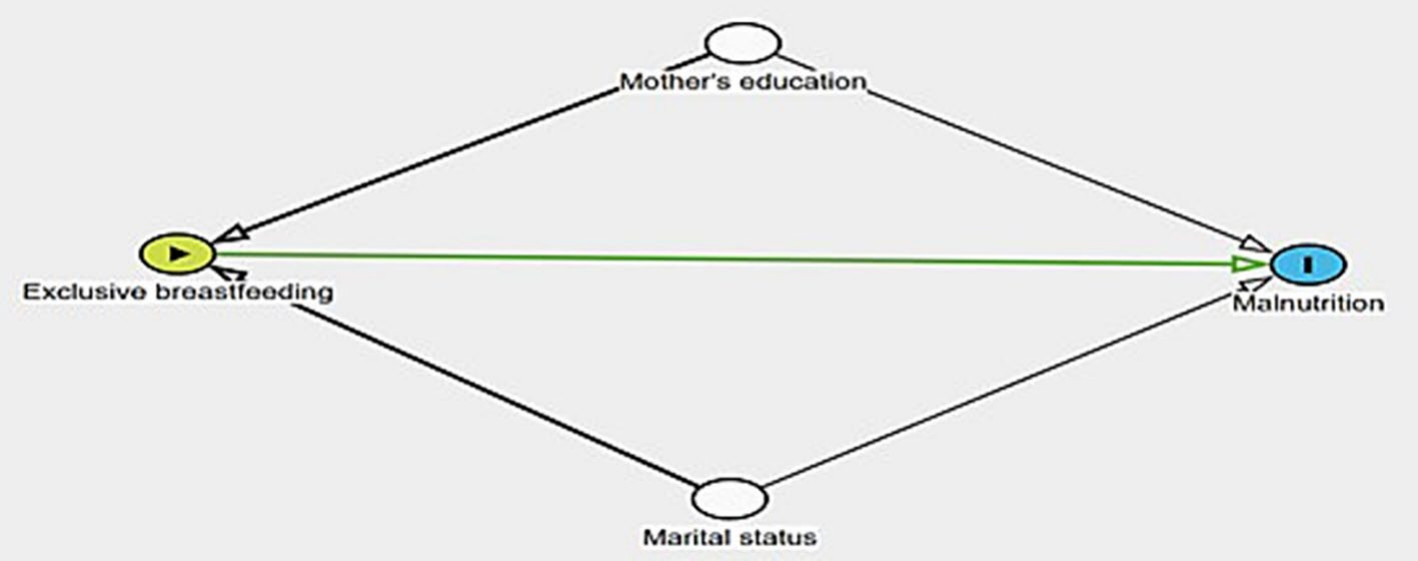
Directed acyclic graph.

Before checking for association, we used a frequentist method to determine effect modifiers and potential confounders. No effect modifier was identified, but rather two potential confounders (mother’s education and marital status). This relationship is presented by the use of a Directed Acyclic Graph (DAG) in Figure 1 that shows that the Education level and Marital status of the mother influence exclusive breastfeeding and malnutrition.

Table 3 presents the results of the BBLR, which used a burn-in of 1,000 iterations and generated 6,000 posterior samples from 3 MCMC chains. After adjusting for potential confounders, the odds of malnutrition among exclusively breastfed babies are 10.36% lower compared to babies who are not exclusively. This relationship is not significant.

**Table 3:** Effect of exclusive breastfeeding on malnutrition.

Adjustment	Predictor	Posterior mean	Odds ratio	Posterior SE	Monte Carlo SE	95% Credible interval of posterior mean
No	Intercept	−0.9644	0.3814	0.1000	0.0013	[−1.1599, −0.7714]
	Exclusive breastfeeding: Yes	−0.1448	0.8652	0.1180	0.0014	[−0.3601, −0.0619]
Yes	Intercept	−0.9756	0.3770	0.1030	0.0013	[−1.1825, −0.7810]
	Exclusive breastfeeding: Yes	−0.1094	0.8964	0.1086	0.0014	[−0.3315, 0.0963]

### 3.3 Final model diagnostic based on Gelman-Rubin, trace, and density plots

**Figure 2:**
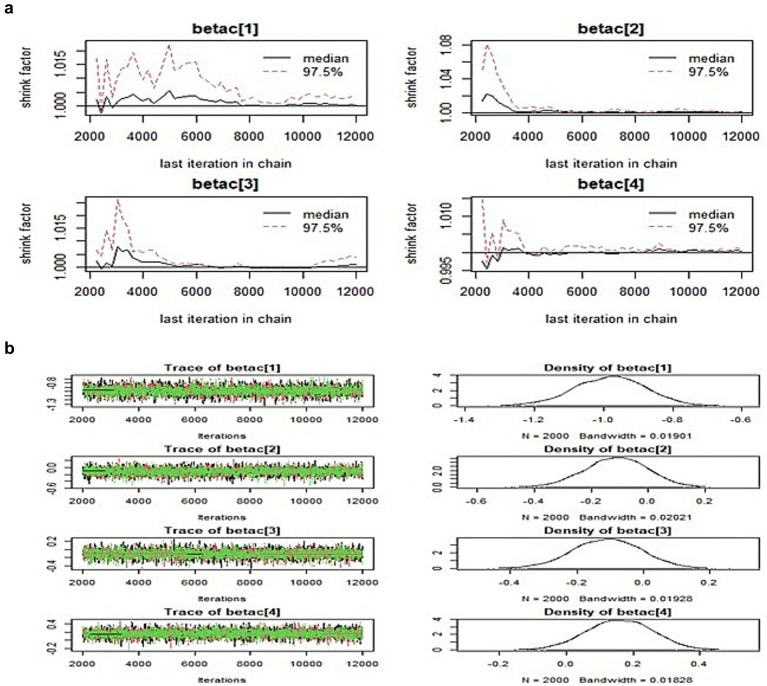
Directed acyclic graph. **(a)** Gelman plots and **(b)** Traceplots and density plots.

The model was assessed and found to have converged and achieved the best fit as showed in Figure 2. The Gelman-Rubin diagnostic plots in Figure 2a show potential reduction factors for all parameters decrease and approach 1 value indicating the between-chain and within-chain variances have become similar. This pattern provides strong evidence that the chains have converged to the same target posterior distribution, supporting the stability of the sampling process and suggesting that the resulting model estimates are reliable. The Figure 2b shows a traceplots and density plots where the traceplots on the left side shows that the Markov chains mix well with stable, overlapping, and patternless trajectories across iterations, indicating that they have reached a stationary distribution and that the model has converged. This suggests that the samples are no longer influenced by initial values and are being drawn from the target posterior distribution. Additionally, the density plots appear smooth and unimodal, further supporting convergence by demonstrating that all chains are sampling from the same underlying distributions. These diagnostics provide strong evidence that the model has converged and that the resulting parameter estimates are reliable.

### 3.4 Study findings from healthcare providers who participated in this study

#### 3.4.1 General characteristics of healthcare providers

The majority of healthcare providers who participated in this study were female (73%, 11/15) and held diploma qualifications (53%, 8/15), with an average age of 38 years, and three-quarters (73%, 11/15) reported 5 years of work experience. In Table 4, most healthcare providers classify themselves as having skills in nutrition, breastfeeding, recognizing signs, and hygiene (93.3–100%). Regarding provision of health education to postnatal women, a considerable number of health providers self-rated good or more competence in nutrition (67%, 10/15), breastfeeding (67%, 10/15), hygiene (60%, 9/15), and fewer recognized signs (33%, 5/15).

**Table 4:** General characteristics of healthcare providers.

Characteristic	Variable	Category	*N*	%	Variable	Category	*N*	%
Skills and competence	Nutrition skills	Yes	15	100.0	Breastfeeding skills	Yes	15	100.0
		No	0	0.0		No	0	0.0
	Hygiene skills	Yes	14	93.3	Awareness of early signs	Yes	15	100.0
		No	1	6.7		No	0	0.0
	Nutrition competence	Poor	2	13.3	Breastfeeding competence	Poor	1	6.6
		Average	3	20.0		Average	4	26.7
		Good	8	53.3		Good	4	26.7
		Very Good	1	6.7		Very Good	6	40.0
		Excellent	1	6.7		Excellent	0	0.0
	Hygiene competence	Poor	2	13.3	Competence in early signs	Poor	3	20.0
		Average	5	33.2		Average	7	46.7
		Good	6	40.0		Good	5	33.3
		Very Good	2	13.3		Very Good	0	0.0
		Excellent	1	0.2		Excellent	0	0.0

#### 3.4.2 Healthcare providers’ practices in delivering health education

In this study, a total of 15 health education sessions were observed. It was observed that during the health education sessions, limited discussions were focused on breastfeeding counseling, assessment of breastfeeding status, guidance on breastfeeding positions, and counseling on exclusive breastfeeding. These findings correspond with provider-reported gaps in reported competences in across different nutritional topics during health education sessions. Hygiene counseling was similarly low discussed, with minimal emphasis on utensil hygiene, safe water, and breast hygiene, which is consistent with mothers’ reported practices, where most did not wash their breasts before breastfeeding. Despite providers reporting relatively good competence in nutrition, observation indicated that counseling on early signs of illness was largely absent, limiting preventive child health education (see Table 5).

**Table 5:** Healthcare providers’ practices in delivering health education (Observation data).

Characteristics	Variable	Category	*N*	%	Variable	Category	N	%
Salutation	Salutation	Yes	15	100	Mentioning aim	Yes	3	20
		No	0	0		No	12	80
	Open question	Yes	3	20	Showing interest	Yes	9	60
		No	12	80		No	6	40
	Avoid judgment	Yes	3	20	Understand	Yes	2	13.3
		No	12	80		No	13	86.7
	Reflection	Yes	3	20	Reminder	Yes	4	13.3
		No	12	80		No	11	86.7
Breastfeeding	Ask breastfeed status	Yes	4	13.3	Exclusive breastfeeding	Yes	6	40
		No	11	86.7		**No**	**9**	**60**
	Breastfeed 8 times	Yes	6	40	Breastfeeding position	Yes	3	20
		No	9	60		**No**	**12**	**80**
	Breastfeed 2 years	Yes	9	60				
		No	6	40				
Hygiene	Clean breast	Yes	4	13,3	Wash utensils after use	Yes	0	0
		No	11	86.7		No	15	100
	Food hygiene	Yes	8	53.3	Safe water	Yes	3	20
		No	7	46.7		**No**	**12**	**80**
	Wash hands after feces	Yes	1	6.7				
		No	14	93.7				
Nutrition	Carbohydrate	Yes	8	53.3	Protein and fibers	Yes	8	53.3
		No	7	46.7		No	7	46.7
	Fats and vegetables	Yes	8	53.3	Fruits and minerals	Yes	8	53.3
		No	7	46.7		No	7	46.7
Signs	Early signs	Yes	2	13.3	Early symptoms	Yes	1	6.7
		No	13	86.7		No	14	93.3

#### 3.4.3 Healthcare providers’ challenges in delivering health education

**Figure 3:**
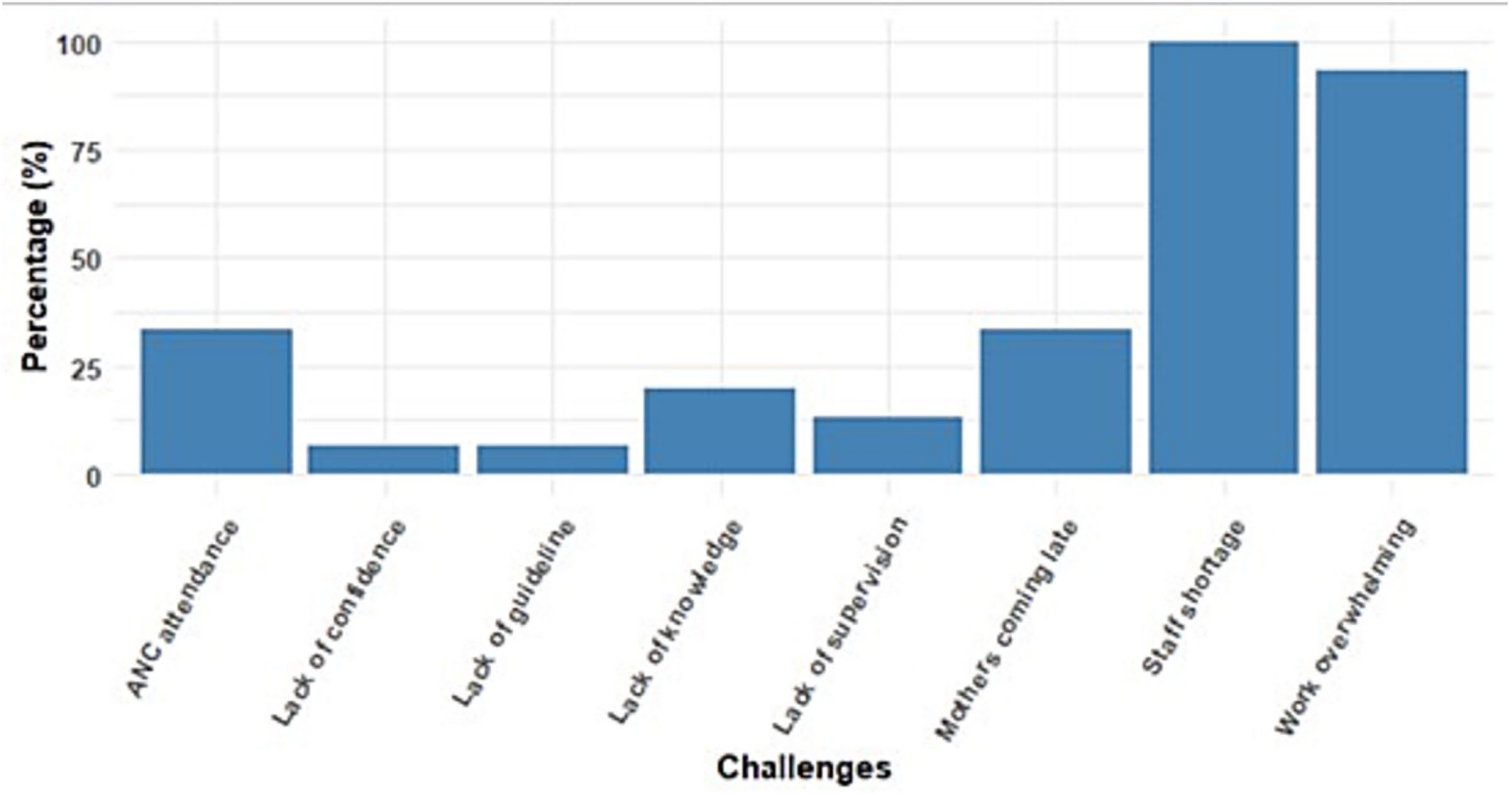
Challenges faced by healthcare providers.

Healthcare providers reported several operational challenges affecting their ability to deliver effective nutritional health education. As illustrated in Figure 3, the most commonly reported challenges included staff shortages, work overload, high antenatal clinic (ANC) attendance, and mothers arriving late to the clinic.

## 4 Discussion

Malnutrition remains a major threat to children’s survival, growth, and overall development, particularly in low- and middle-income countries (2). In this study, the overall prevalence of malnutrition was 27.8%, with 16.4% stunting, 7.8% wasting, 3.6% underweight, and 4.0% overweight. Exclusive breastfeeding was low at 18%, and although exclusively breastfed children had lower odds of malnutrition, the association was not statistically significant after adjustment for maternal education and marital status. Findings from healthcare providers and observations help explain these results, as providers had good knowledge, but important gaps were seen in practice, especially in breastfeeding support and breast hygiene counseling. Similarly, mothers reported low exclusive breastfeeding and poor breast hygiene practices. Overall, limited practical counseling on breastfeeding and hygiene at the facility level may have contributed to suboptimal feeding practices, which is reflected in the observed burden of malnutrition among children.

Building on the overall findings, the prevalence of malnutrition in this study (27.8%) is slightly lower than the 30.49% reported among children aged 0–23 months in Tanzania, where malnutrition was defined as experiencing at least one form of stunting, wasting, or underweight (23). This similarity suggests that a substantial proportion of young children experience at least one form of malnutrition across different settings. However, the lower prevalence observed in this study may be due to differences in the study setting, as the present study was conducted among children attending health facilities, while the comparison study used a broader population. From a public health perspective, these findings are important because malnutrition during the first 2 years of life can lead to poor growth, delayed development, increased risk of infections, and long-term health problems. Therefore, this study findings will help in improving early interventions to improve child nutrition.

When examining individual forms of malnutrition, stunting was the most common condition in this study, followed by wasting and underweight. Within Tanzania, the prevalence of stunting observed in this study (16.4%) is lower than the 30% reported in the Tanzania Demographic and Health Survey, while wasting (7.8%) is higher than the national estimate of 3%, and underweight (3.6%) is lower than the reported 12% (4). Similarly, the overall burden of malnutrition in this study is lower than findings from another study in Tanzania, reporting 49% of children experiencing at least one form of malnutrition, including higher levels of stunting (43%) and wasting (7.4%) (14). These differences may be due to variations in study setting, age groups, and access to healthcare services, since this study was conducted in health facility settings where children may benefit from regular growth monitoring and nutrition counseling. Beyond Tanzania, the prevalence of stunting in this study is lower than that reported in Kenya (23%) and comparable to findings from Indonesia (14.1%), while wasting is similar to Kenya (6%) but lower than levels reported in some Sub-Saharan African countries, such as Nigeria (16.6%) and Benin (16.4%). In contrast, the underweight prevalence in this study is lower than that reported in countries such as Chad (32.5%) and Niger (35.5%) (24–26). These variations highlight the influence of factors such as food security, maternal and child health practices, and public health interventions. Overall, the findings show the need for strategies that fit local settings to improve early child nutrition, especially in the community, while maintaining the progress made through health facility services.

To reduce the risk of malnutrition, exclusive breastfeeding during the first 6 months is recommended by the WHO. In this study, the prevalence of exclusive breastfeeding was low (18%), which is below the recommended level and lower than what has been reported in some other settings. Children who were exclusively breastfed had lower odds of malnutrition; however, this association was not statistically significant after adjusting for confounders. Similar non-significant findings have been reported in some studies, while studies in India and Tanzania have shown a significant protective effect of exclusive breastfeeding on child nutritional outcomes (27–29). The lack of statistical significance in this study may be explained by the low prevalence of exclusive breastfeeding, differences in how exclusive breastfeeding was measured, and the use of overall malnutrition as an outcome rather than specific indicators such as stunting, wasting, or underweight. Overall, these findings suggest that strengthening breastfeeding counseling and support at health facilities is important to improve exclusive breastfeeding practices and may contribute to reducing child malnutrition.

Building on the findings above, maternal education and marital status were included as confounding factors in this study because they can influence both breastfeeding practices and child nutritional status, and therefore may distort the observed association if not properly adjusted for. Mothers who were married may benefit from greater social and financial support, shared childcare responsibilities, and encouragement from partners, which can make it easier to practice exclusive breastfeeding and ensure better child feeding practices. Similarly, mothers with higher education levels are more likely to have better health literacy, understand recommended infant feeding practices, and access health information, which can improve breastfeeding behavior and child nutrition. In this study, both marital status and education were associated with lower odds of malnutrition and influenced exclusive breastfeeding practices, indicating their confounding role. If these factors were not considered, the relationship between exclusive breastfeeding and malnutrition could be masked or misestimated. These findings are consistent with evidence from India, where maternal characteristics such as education and related socio-demographic factors were also shown to influence infant feeding practices and child nutritional outcomes (28).

Furthermore, this study integrated findings from healthcare providers, observations, and mothers to better understand the persistence of malnutrition. Although healthcare providers reported good knowledge of nutrition, breastfeeding, and hygiene, observations showed that breastfeeding counseling was often incomplete, with limited assessment of breastfeeding practices, poor guidance on positioning, and little emphasis on exclusive breastfeeding. This is similar to findings from a qualitative study in Tanzania, which reported poor support from healthcare providers, limited education on exclusive breastfeeding, and a misunderstanding of the definition of exclusive breastfeeding among mothers, leading to low exclusive breastfeeding practice (30). A quantitative study in Tanzania also reported gaps in nutrition knowledge among nurses in remote areas, particularly on appropriate child feeding practices, such as when to introduce solid foods (31). Hygiene counseling in the present study was relatively better, but little attention was given to breast hygiene. These gaps were reflected in mothers’ practices, where although many reported boiling water and washing utensils, most did not wash their breasts before breastfeeding, and exclusive breastfeeding remained low. Overall, these consistent findings across data sources and studies suggest that gaps in the quality of breastfeeding counseling at health facilities contribute to suboptimal infant feeding practices, which may help explain the continued burden of malnutrition in Tanzania and similar settings.

Nevertheless, this study has some limitations. It was conducted only among mothers attending RCH clinics, so the findings may not represent all mothers in the community. Also, because it is an observational study, it cannot establish a causal relationship between breastfeeding and malnutrition. In addition, the number of healthcare providers observed was small, which may limit how widely the findings on provider practices can be applied.

Despite these limitations, the study has important strengths. Exclusive breastfeeding was measured using a strict WHO definition derived from multiple questions, not only a 24-h recall, which improves accuracy. The study also used triangulation of three data sources (healthcare providers, direct observation, and mothers with their children) collected simultaneously and in the same place, providing a fuller picture of practices and outcomes. In addition, Bayesian regression with non-informative priors and credible intervals was used for analysis, providing reliable estimates. Finally, this is among the first studies to assess all four forms of malnutrition together in this setting.

In conclusion, this study observed lower odds of malnutrition among exclusively breastfed children compared with those who were not exclusively breastfed. However, the association was not statistically significant after adjustment for maternal education and marital status. The prevalence of exclusive breastfeeding among mothers attending the selected health facilities was also relatively low. The findings further showed that healthcare providers had adequate knowledge on nutrition, breastfeeding, hygiene, and early signs of illness, but their delivery of both individual and group health education during postnatal and child health services was limited due to challenges such as staff shortages, heavy workload, and high patient numbers.

To address these gaps in a low-resource setting, counseling can be strengthened by integrating and rotating health education duties among available staff, rather than requiring additional personnel, and by incorporating refresher training into existing health programs to improve counseling skills. At the community level, community health workers can be engaged to reinforce key messages on exclusive breastfeeding, hygiene, and child feeding practices during home visits and outreach activities, which is a feasible and widely used strategy in similar settings. Strengthening both facility-based counseling and community follow-up within existing health system structures may improve maternal practices and contribute to better child nutritional outcomes.

## Data Availability

The raw data supporting the conclusions of this article will be made available by the authors, without undue reservation.
